# Y Chromosome Story—Ancient Genetic Data as a Supplementary Tool for the Analysis of Modern Croatian Genetic Pool

**DOI:** 10.3390/genes15060748

**Published:** 2024-06-06

**Authors:** Dragan Primorac, Jelena Šarac, Dubravka Havaš Auguštin, Natalija Novokmet, Tamer Bego, Ron Pinhasi, Mario Šlaus, Mario Novak, Damir Marjanović

**Affiliations:** 1 St. Catherine Specialty Hospital, 10000 Zagreb, Croatia; 2 Faculty of Dental Medicine and Health, Josip Juraj Strossmayer University of Osijek, 31000 Osijek, Croatia; 3 School of Medicine, Josip Juraj Strossmayer University of Osijek, 31000 Osijek, Croatia; 4 Medical School, University of Split, 21000 Split, Croatia; 5 Department of Biochemistry & Molecular Biology, The Pennsylvania State University, State College, PA 16802, USA; 6 The Henry C. Lee College of Criminal Justice and Forensic Sciences, University of New Haven, West Haven, CT 06516, USA; 7 Regiomed Kliniken, 96450 Coburg, Germany; 8 Medical School, University of Rijeka, 51000 Rijeka, Croatia; 9 National Forensic Sciences University, Gandhinagar 382007, India; 10 Centre for Applied Bioanthropology, Institute for Anthropological Research, Gajeva 32, 10000 Zagreb, Croatia; 11 Faculty of Pharmacy, University of Sarajevo, 71000 Sarajevo, Bosnia and Herzegovina; 12 Department of Evolutionary Anthropology, University of Vienna, 1030 Vienna, Austria; 13 Human Evolution and Archaeological Sciences (HEAS), University of Vienna, 1030 Vienna, Austria; 14 Anthropological Center, Croatian Academy of Sciences and Arts, 10000 Zagreb, Croatia; 15 Department of Archaeology and Heritage, Faculty of Humanities, University of Primorska, 6000 Koper, Slovenia; 16 International Burch University, 71000 Sarajevo, Bosnia and Herzegovina; 17 Faculty of Biotechnology and Drug Development, University of Rijeka, 51000 Rijeka, Croatia

**Keywords:** Y chromosome, SEE, Croatia, modern genetic data, ancient genetic data

## Abstract

Due to its turbulent demographic history, marked by extensive settlement and gene flow from diverse regions of Eurasia, Southeastern Europe (SEE) has consistently served as a genetic crossroads between East and West and a junction for the migrations that reshaped Europe’s population. SEE, including modern Croatian territory, was a crucial passage from the Near East and even more distant regions and human populations in this region, as almost any other European population represents a remarkable genetic mixture. Modern humans have continuously occupied this region since the Upper Paleolithic era, and different (pre)historical events have left a distinctive genetic signature on the historical narrative of this region. Our views of its history have been mostly renewed in the last few decades by extraordinary data obtained from Y-chromosome studies. In recent times, the international research community, bringing together geneticists and archaeologists, has steadily released a growing number of ancient genomes from this region, shedding more light on its complex past population dynamics and shaping the genetic pool in Croatia and this part of Europe.

## 1. Introduction

Molecular anthropology could be defined as a branch of anthropology that studies the origin, kinship, history, and migration patterns of human populations at the molecular level, based on human genetic polymorphisms. This term was introduced by Emil Zuckerkandl half a century ago, when he described molecular anthropology as a scientific discipline that deals with studying primate phylogeny and human evolution through the study of genetic information written in proteins and polynucleotide molecules [1]. Today, molecular anthropology is focused on the primary carrier of hereditary information (DNA), and its application is more significant and broader than Zuckerkandl imagined.

Our views of the past and especially of European prehistory have been reshaped in the last few decades by molecular anthropology, mostly by data obtained from uniparental genetic systems—mitochondrial DNA and Y chromosomes. Twenty years ago, Mark Jobling described the Y chromosome as a classic juvenile delinquent who refuses to socialize with others, is full of “garbage”, contains very few genetically functional segments, and unstoppably tends towards evolutionary degeneration. Therefore, it is not surprising that “non-recombinant desert” and “gene-poor chromosome” are examples of the different descriptions used for the Y chromosome in the last few decades [2]. However, the Y chromosome is considered one of the most population-genetically informative systems because it has the largest non-recombining block (95% of its length) compared to all other chromosomes in the human genome. This non-recombining block represents a significant source of information in the reconstruction of the evolutionary past of human populations, monitoring the migration flows of men in different periods of the past. Namely, men tend to migrate over longer distances than women, and due to the widespread practice of patrilocality, men typically reside closer to their birthplace, while women exhibit higher levels of migration at the local level. Consequently, the Y chromosome displays more pronounced local differentiation, while mtDNA is more uniformly distributed across populations and demonstrates a significantly lower degree of geographic clustering [3].

Archaeological findings indicate that the territory of modern Croatia has been continuously settled since the Paleolithic, with some of the crucial Neandertal sites such as Vindija and Krapina [4,5]. It was also one of the first areas in Europe affected by migrations from Anatolia, which was associated with the spread of farming during the early Neolithic (about 6000 BCE) [6]. The first pastoralists arrived in these parts following major rivers such as the Danube but also using the Adriatic coastal route, confirmed by recent paleogenetic studies [7,8]. A distinct change in settlement patterns, material culture, social traditions, and subsistence practices came with the Eneolithic period (Copper age) in the region [9]. It was also characterized by one of the earliest utilizations of metal (copper) objects in Europe, the emergence of important pan-regional archaeological cultures such as the Vučedol culture, and by the first evidence of violence on a massive scale [10,11]. The beginning of the Bronze Age in Europe, as well as in its southeastern part, was characterized by the significant impact of the Yamnaya people, in both genetic and linguistic terms [12,13,14,15], as well as with the introduction of previously unknown infectious diseases such as plague [16]. As a result of the fusion of Yamnaya-related ancestry with the already existing Balkan Neolithic populations during the Bronze Age, we can observe the formation of the cultural groups (the alleged “proto-Illyrians”) that are well known in the Iron Age under different names, such as Iapodes, Delmatae, and so on.

**Figure 1:**
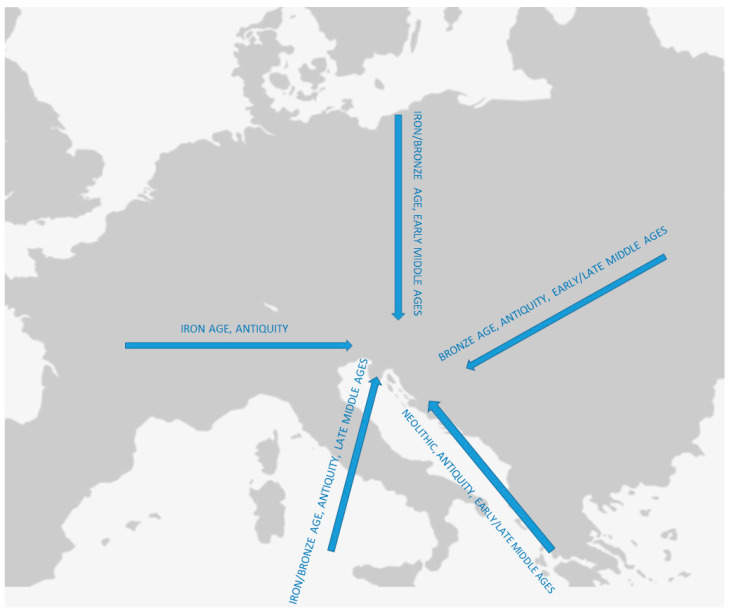
Proposed historic migration episodes in Southeastern Europe since the Neolithic period.

The period of Roman dominance that started at the turn of the era is one of the key epochs in the historical development of this part of Europe. The process of Romanization involved urbanization and cultural assimilation into the Roman way of life, accelerating technological development, centralization of the state, construction of roads and complex transportation systems, the establishment of a permanent army and administration, economic expansion, and growth of trade. All this resulted in profound social, cultural, and population changes on a local and regional scale, some of which are still visible today [17,18,19,20]. However, from the 3rd century CE, beginning with the so-called “3rd-century crisis”, the Roman state experienced profound political instability and several military and economic upheavals that, in combination with pandemics and environmental changes [21], resulted in the mass movement of people across the continent (Great Migration Period, 4th–8th century CE). These migrations and resulting changes were not a short-term sudden event. Still, they involved a set of long-term processes that had a significant impact on all aspects of life, primarily affecting demography and genetic structure. In SEE, these processes involved the migration and settlement of various groups of different origins, such as Germanic (Langobards, Ostrogoths, Gepids), Asian populations (Huns and Avars) [22,23,24], and the Slavs. The migration of the Slavs, whose impact shaped the linguistic landscape of the Balkans with the widespread usage of South Slavic languages seen today, has recently been documented through paleogenetic research [19]. Finally, there are assumptions that the expansion of the Ottoman Empire during the late medieval and early modern periods in this part of Europe also initiated several important local migrations (Figure 1).

Previously published molecular genetic analysis of the genetic pool of the modern SEE male population confirmed the extraordinary heterogeneity and complexity of this population as well as its migrations. It confirmed that there was a high degree of mixing of the newly arrived settlers with the indigenous populations that had already been present in the region. As illustrated in this review, earlier research offered a genetic scenario for the most important migration episodes that strongly influenced the peopling process of the territory of modern Croatia. Most of these studies investigated the ancestral genetic impact of Old Europeans and Early Farmers on Croatians. They proved that the Croatian population, like almost any other European population, represents a remarkable genetic mixture. The latest ancient DNA analyses are opening interesting new insights in this context and initiating possible restructuring of existing, published scenarios of the peopling of SEE.

## 2. Modern Y-Chromosome Diversity of Southeastern Europe

A considerable number of population-genetic studies have, in the last few decades, provided a large amount of Croatian and other SEE population data based on Y-chromosome markers. The data reflect the turbulent and complex demographic history of this region, influenced by gene flow from various parts of Eurasia and a long history of intermixing [25,26,27,28,29,30,31,32,33,34,35,36,37,38,39]. Studies have confirmed a high level of Y-haplogroup and -haplotype diversity in SEE, with three NRY haplogroups accounting for the majority of paternal genetic variation—namely the I2a, R1a, and E1b clades [38,39]. These three haplogroups account for >70% (40%, 20%, and 10%, respectively) of the paternal gene pool in SEE, with several other lineages present in lower percentages (R1b, G2a, J1, and J2). 

### 2.1. Early Studies of Y-Chromosome Markers

A pioneering study on Y-chromosome markers in SEE was conducted by Semino and colleagues at the beginning of the 21st century [26]. Those preliminary studies were subsequently expanded [28]. Their phylogenetic analyses unveiled a remarkably high frequency of the M170 mutation (mostly present, nowadays, in haplogroup I2a), pinpointing the Adriatic coast as the likely post-glacial origin of this mutation, from where it spread across Southeastern and Southern Europe. In Croatia, Y-chromosome haplogroups characteristic of Western, Southern, and Eastern Europe were identified, alongside a prevalence of Paleolithic origin markers and a scarcity of Neolithic markers specific to the Mediterranean region of Europe. As the exploration of new markers progressed, a subsequent study analyzed high-resolution Y-chromosome data from seven populations in Southeastern Europe, offering further insights into the genetic contribution of Southeastern European inhabitants to the modern European gene pool [30]. While typical European haplogroups were present in Southeastern European populations, their distribution and estimated ages underscored the region’s significance in shaping the geographical structure of the European paternal gene pool.

### 2.2. Comprehensive Reviews and Recent Genetic Contributions

**Figure 2:**
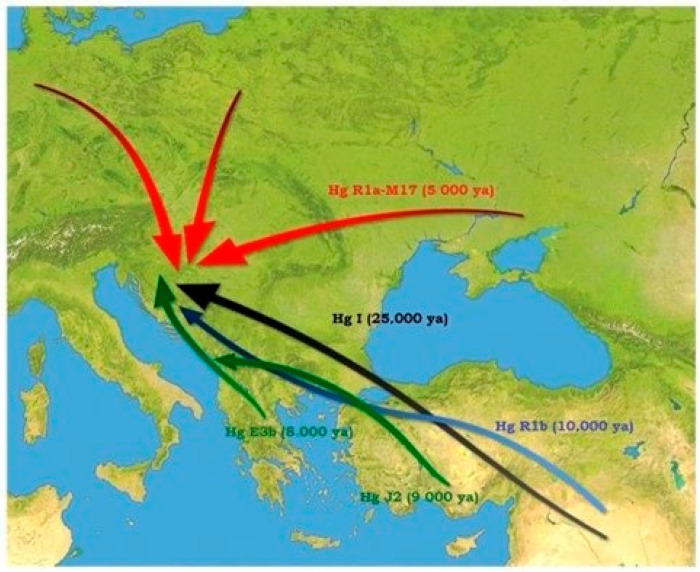
Proposed migration routes for the main observed haplogroups in the modern Croatian population [36].

In 2011, Primorac and colleagues published a comprehensive review based on population genetic findings published up to this point (Figure 2). The review suggested that around three-quarters of present-day Croatian men most likely trace their ancestry back to ancient Europeans who settled in the region both before and after the last glacial maximum. The remaining population was believed to be descendants of individuals who migrated to this area via the southeastern route over the past 10,000 years, primarily during the Neolithization process [36].

A more detailed analysis revealed a rich diversity of haplogroups, bearing distinct signals indicative of three major historical waves of settlement in the region—haplogroups I2a, R1a, and E1b [38]. This study reaffirmed and expanded previous findings, shedding more light on significant (pre)historical movements reflected in Croatia’s paternal genetic heritage. Although it was, until then, suggested that the prevalent haplogroup I2a (represented with 30–40% in SEE ) had a substantial role in the SEE gene pool during the Upper Paleolithic era, more recent research indicated that most paternal lineages have been notably influenced by more recent and rapid demographic changes over the last 5000 years [40]. Thus, Šarac and colleagues emphasized that certain haplotypes might have been introduced into the SEE gene pool at different periods, some even recently [38].

**Figure 3:**
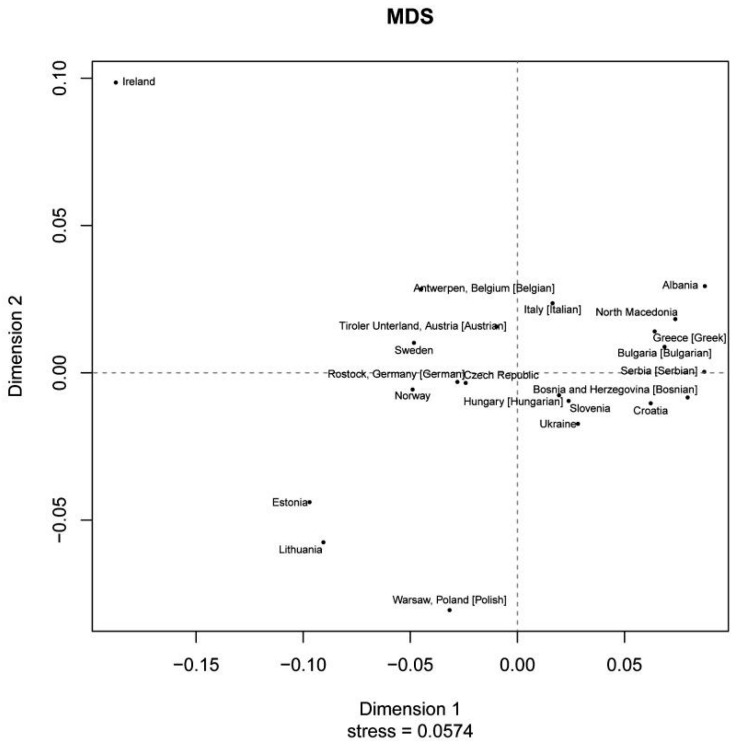
Multidimensional Scaling (MDS) plot showing genetic similarities between different SEE countries based on Y chromosome [39].

In a study published in 2022, Primorac and colleagues continued their exploration of Croatia’s paternal genetic heritage [39]. An interpopulation study utilizing 17 Y-STR markers revealed the lowest genetic diversity between the Croatian population and its neighboring Bosnian-Herzegovinian population, with haplogroup I2a being the most prevalent (Figure 3). The second most prevalent haplogroup was R, primarily represented by its major sublineage R1a, except in the Hvar subpopulation, where E1b emerged as the second most prevalent haplogroup. Rare haplogroups, including L, T, and Q, were also confirmed in this study, with G1 being detected for the first time in the Croatian population. Once again, it was proposed that most Croatian men (78%), characterized by haplogroups I, R1a, and E1b, carry the ancestral genetic legacy of Old European populations. The remainder of the population was believed to have descended from individuals who arrived primarily during the Neolithization process [39].

In other parts of SEE, such as Serbia, Scorrano and colleagues (2017) [41] also noted the predominant prevalence of paternal lineage I2a (42%), followed by haplogroups R1a at 15% and E1b at 12%, which aligns with research findings in Bosnia and Herzegovina. Namely, Marjanović and colleagues conducted seminal research on the settlement history of Bosnia and Herzegovina in 2005. They utilized Y-chromosome analyses and categorized participants into three ethnic groups based on the origin of their paternal grandfathers [29]. Haplogroup I emerged as the most prevalent haplogroup, exceeding (for the population of modern Bosnia and Herzegovina, including results for three main ethnic groups) 50%, while other haplogroups, such as E1b and R1a, exhibited frequencies above 10%. Despite the observed genetic differences among the ethnic groups, they did not reach statistical significance, indicating a genetically cohesive population. Marjanović underscored that although Bosnia and Herzegovina is globally recognized for the high frequencies of haplogroup I2a, the implications of its high frequency remain uncertain. It could be explained by the favorable ecological conditions during the last Ice Age in SEE that enabled the survival of I2a through harsh winters. Still, it could also be associated with more recent migrations. Marjanović also highlighted that the R1a haplogroup could not be exclusively designated as a “Slavic marker,” given its presence both before and after the arrival of the Slavs in SEE [42].

### 2.3. Settlement Patterns and Genetic Transitions in SEE

In the context of genetic investigations into the SEE settlement patterns, it is pertinent to highlight the study conducted by Kovačević and collaborators in 2014. Through meticulous analyses of samples originating from the Western Balkans region, encompassing Croatia, Serbia, Bosnia and Herzegovina, Macedonia, Montenegro, and Kosovo, researchers illustrated that the genetic composition of the examined populations denotes a subtle transition between Southern and Central European populations. The results revealed an exceptionally high genetic similarity among populations speaking languages from the Slavic language family. The sole noteworthy deviation was observed in the Albanian population from Kosovo, consistent with earlier analyses of uniparental and STR markers. Each analytical approach distinctly portrayed the “geographical position” of the populations under study. Consequently, the overarching conclusion drawn from this study is that the autosomal outcomes aligned with prior findings derived from uniparental markers, mtDNA, and the Y chromosome. Furthermore, the study underscored that populations in this region genetically mirror a distinct (pre)historical convergence point between the Middle East and Europe [43].

### 2.4. Dominant Y-Chromosome Haplogroups in Modern SEE Population

#### 2.4.1. Haplogroup I2a

Y haplogroup I2a-M423, a marker of Paleolithic European ancestry, predominates in modern SEE populations, particularly in Bosnia and Herzegovina (around 50% for the population of modern Bosnia and Herzegovina, including results for three main ethnic groups, Croats ~71%, Bosnians ~44%, and Serbs ~31%)), Croatia, and Serbia (around 40%) [29,38,41]. Its prevalence gradually diminishes towards the northwest and southeast, suggesting it has endured the challenges of the last Ice Age within refugial areas across SEE and the Balkan Peninsula [44]. Namely, it was initially believed that during the last Ice Age, populations migrated from the northern to the southern regions of the continent, seeking refuge in milder climates during severe conditions (25,000 to 15,000 years ago) and that many settled in refugial centers, including those on the Iberian and Balkan Peninsula and present-day Ukraine. With the ice retreat, these populations then recolonized Central, Eastern, and Northern Europe after the climate stabilized. Haplogroup I2a was identified as a marker of one such expansion from the Balkan Peninsula to other parts of the continent [36,38,44]. However, subsequent research based on the analysis of whole Y chromosomes opened the possibility that this Paleolithic European marker might have arrived in these regions during later migrations associated with the influx of different tribes [40]. Proponents of this theory suggest that the high frequency of this haplogroup in this region may result from the founder effect, wherein a relatively restricted genetic pool led to the dominance of this haplogroup during the formation of new generations.

#### 2.4.2. Haplogroup R1

Haplogroup R emerged as the genetic lineage initiating migration into Europe due to expansion from a broader Eurasian territory [26]. Serving as the ancestral source for prominent Western and Central European haplogroups—R1a and R1b—haplogroup R significantly shapes the genetic makeup of the continent. The origin of the R1 haplogroup can be traced back to Northeast Asia, preceding the onset of the last Ice Age—the examination of skeletal remains of a four-year-old boy, dubbed the Mal’ta boy, discovered in the Altai region of Siberia, dating back approximately 24,000 years, has confirmed the presence of this haplogroup in Central Asia [45]. The dominance of the R1 haplogroup seems prominent in early Indo-European societies, with its expansion intricately linked to the Bronze Age. Notably, carriers of the R1b haplogroup tended to cluster in Central and Western Europe, while those of the R1a haplogroup progressively asserted influence over the Eastern and Southeastern European areas [46,47]. However, these migratory patterns likely unfolded in different waves rather than as singular, unidirectional events. The prevalence of haplogroup R1a is most prominent in Poland (over 55%), European parts of Russia and Belarus (between 45 and 60%), Ukraine and Slovakia (over 40%), the Czech Republic (over 30%), and Austria, Croatia, and Serbia (around 25%), with Bosnia and Herzegovina recording approximately 15% carriers [38,48].

Interestingly, the distribution of this haplogroup correlates with the dispersion of the Balto-Slavic and Indo-Iranian linguistic groups, indicating a significant intersection of linguistic and genetic expansions. Regarding their introduction to SEE, studies propose that R1a lineages arrived from the northern regions of Eastern Europe as part of a broader process, associated with the Corded Ware cultural diffusion during the Copper and Bronze Ages (approximately 5200–4300 years ago), encompassing Slavic expansions [47]. Archaeological findings support the thesis that the Corded Ware tradition emanated from Central Europe, spreading eastward to the Volga, as Underhill reports. Consequently, this haplogroup is colloquially referred to as the “Slavic” haplogroup due to its various branches being associated with the migrations of Slavic tribes in Eastern and Northeastern Europe and the Balkans. In contrast to R1a, haplogroup R1b dominates in Northwestern and Western Europe, reaching frequencies of 80% in Scotland and 90% in the Basque population [45]. Research based on ancient DNA confirms its origin in a location similar to that of the R1a haplogroup, identified as the Proto-Indo-European homeland, between the Black Sea’s northern shores and the Caspian Sea [8,12]. Concerning its presence in Croatia, Myres’ data indicate that the local variant of R1b in these areas lacks a mutation linking it to ancestors from the Iberian Peninsula [46]. Instead, it likely arrived from the Western Asian region, probably through the Levant (modern-day Lebanon), independent of Neolithic farmers. 

#### 2.4.3. Haplogroup E

The phylogenetics of haplogroup E has been the subject of ongoing investigation by various research groups. Recent studies, however, propose its emergence approximately 60,000 years ago within the African continent [49]. In Europe, it is represented by its sub-branch E-V13, prominently observed in Kosovo (approximately 45%), Albania, Montenegro (around 30%), and Serbia (about 20%) and with lower prevalence in other European regions, including Croatia (approximately 10%) [26,29,38]. Initial studies suggested that the mutation V13 was introduced into the Balkans alongside early farming technologies [27]. However, Battaglia et al. (2009) proposed an earlier arrival of this marker in Europe during the late Mesolithic period, followed by a Neolithic dispersal associated with the spread of farming [33]. Some scholars suggested that E1b-V13 may reflect Greek colonization’s demographic and socio-cultural impact [32]. However, an explanation based on ancient DNA research implies that E-V13 potentially developed in Central or Eastern Europe during the Neolithic period and attained significant prevalence in SEE during the Bronze Age [14,19,50].

#### 2.4.4. Haplogroup G and J 

Two Y-chromosome haplogroups are directly linked to Neolithic expansions in Europe. Haplogroup G, emerging approximately 50,000 years ago, underwent evolution in isolation within the Middle East. It is present in European populations through its sub-branch G2a, primarily concentrated in the Mediterranean basin [51]. The frequency (up to 10%) and distribution of this haplogroup, combined with analyses of archaeological skeletal remains, associate it with early Neolithic farmers who migrated to European territories, introducing agriculture to the indigenous “Paleolithic” European gatherers and hunters [45]. Haplogroup J, originating approximately 48,000 years ago, is closely related to the European haplogroup I. While haplogroup J1 entered Europe through Anatolia during the Neolithic expansion, haplogroup J2 originated in the Middle East. It was notably widespread in these regions during the Mesolithic, similar to the carriers of haplogroup E-V13 mentioned previously [45].

The relatively low frequency of haplogroups I2a and E-V13 in regions regarded as of Neolithic origin, such as the Middle East (present-day Turkey), Mesopotamia (present-day Iraq and Syria), Persia, or the Levant (present-day Syria, Lebanon, Israel, Palestine, and Jordan), suggests that these haplogroups are characteristic of Mesolithic hunter–gatherers (indigenous people from SEE) who did not migrate from the Middle East during the Neolithic. Over time, these transformed hunters and gatherers transitioned into farmers, playing a crucial role in the northward diffusion of agriculture. Also, the diminishing Neolithic signal in populations west of Croatia implies that these regions served as a boundary where the migration-driven model of agricultural expansion gradually gave way to the dissemination of the farming concept among the local population [38,42]. 

## 3. Ancient Y-Chromosome Diversity of Croatia and the Neighboring Populations

In the last decade, advancements in molecular biology technology have facilitated the isolation and sequencing of DNA extracted from ancient remains, thereby deepening our understanding of past populations and revealing intriguing and sometimes contradictory insights, especially regarding the most common SEE Y haplogroup—I2a. Ancient DNA proof confirmed haplogroup I as one of the oldest European haplogroups—it has been detected in Palaeolithic hunter–gatherers from Switzerland, Hungary, and Scandinavia, as well as in Neolithic and Bronze Age samples from Hungary, Germany, and Iberia [8,12,14,52,53,54,55,56]. For example, complete NRY sequences of five male individuals (one from Luxemburg and four from Sweden), dated to 8 kya, showed that all five belonged to the I haplogroup [53]. In the SEE context, the I2a lineage typical for this region has been found in a Croatian site belonging to the Neolithic Starčevo (5500 and 4500 BCE) [8,57]. A study from 2021 also detected two I2a1 individuals belonging to the Middle Copper Age Lasinja culture in continental Croatia [11], while Lazaridis and colleagues also reported three individuals in Romania and one in Bulgaria [14]. This confirms its long-term presence in the region; however, it is significantly lower in prevalence than is evident today, which is puzzling.

The most recent archaeogenetic studies involving SEE (including Croatia) suggested that SEE was a vital link between the Near East and the rest of Europe in (pre)historical times [14,19]. The authors state that more than half of the autosomal ancestry of Southeast Europeans today comes from the mentioned Slavic migrations, based on the analysis of autosomes, and that modern-day Croats are 66.5% of Central-Eastern European early medieval Slavic ancestry, 31.2% of local Roman ancestry, and 2.4% of West Anatolian-Ottoman ancestry [19]. When looking at the paternal genetic ancestry reported in the study, Olalde and colleagues assert that the genetic lineages I2a and R1a consistently exhibit associations with Eastern European ancestry within the autosomes. They propose that these lineages were introduced to the Balkans by Slavic migrants from Eastern Europe during the Early Medieval period. However, out of 78 male individuals reported in the study (covering a significant historical period, from the 1st to the 15th century CE), only 4 unrelated I2a1 individuals from different Serbian sites were identified, dating to later, different medieval periods (8th–14th century CE). Given the significance of Slavic migrations for this region, one would expect a higher prevalence in early medieval times if the I2a is to be perceived as a “Slavic” signal.

**Figure 4:**
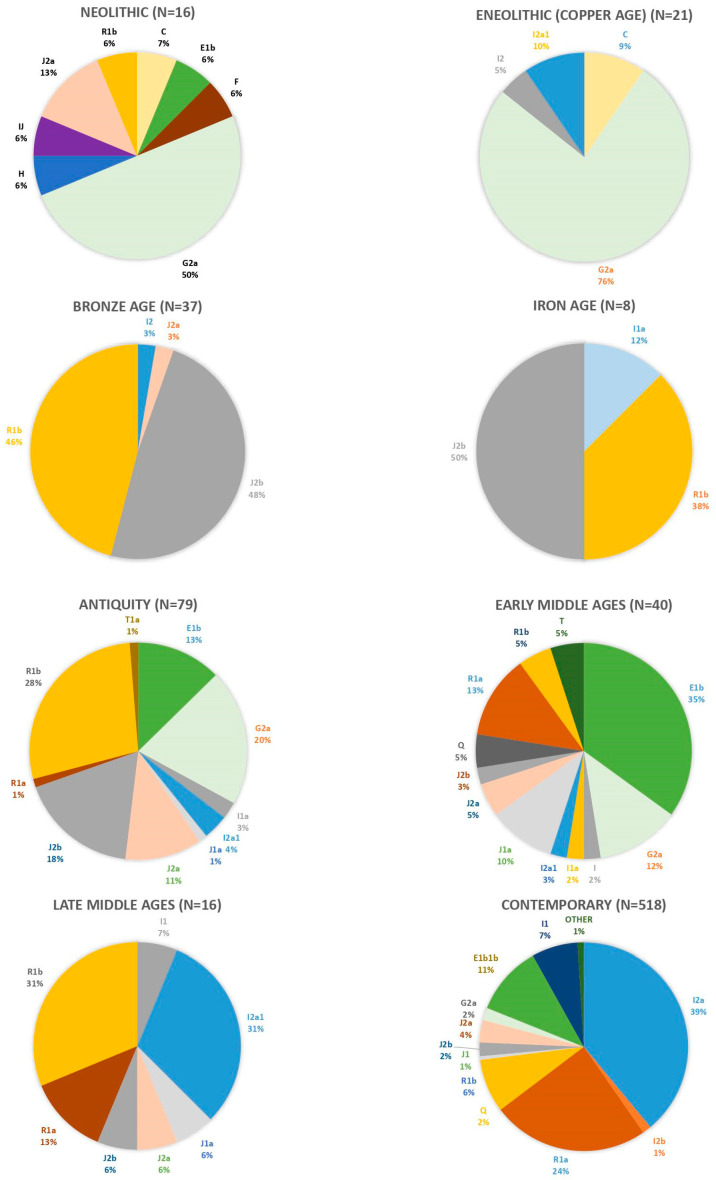
Y-chromosome haplogroup distribution in SEE from Neolithic to contemporary period based on ancient ([8,11,14,15,19], Pinhasi R. and Novak M., personal communication) and modern genomes [39].

Furthermore, published and unpublished aDNA data from Croatia, spanning a period from the Neolithic to the Middle Ages and comprising more than 200 male individuals, reveal a general scarcity of the I2a lineage (Pinhasi R. and Novak M., personal communication). This finding presents a partial challenge to the hypothesis formulated from studying the contemporary Croatian population. Although the sample size is limited and distribution across historical periods is somewhat unbalanced, it shows interesting patterns that could diverge to a certain extent from the commonly accepted views regarding the shaping of the paternal landscape in SEE (Figure 4).

Namely, the predominant haplogroup identified in analyzed Neolithic sites (ca 6000–4500 BCE; Starčevo, Sopot and Lengyel archaeological cultures in continental Croatia, Cardium impress, Hvar and Danilo cultures in the Adriatic region) was G2a, representing 50% of the male samples, which was expected. Throughout the Eneolithic period (ca 4500–2500 BCE), this pattern persisted, but during the subsequent Bronze (ca 2500–750 BCE) and Iron Ages (ca 750 BCE—1 CE), two lineages, namely J2b and R1b, became dominant. The paternal genetic landscape experienced notable shifts during Antiquity (ca 1–500 CE) in terms of diversity and haplogroup distribution. In addition to J2b and R1b, two other haplogroups, E1b and J2a, emerged with frequencies exceeding 10%. This observed rise in genetic diversity correlates with increased population movements into SEE during the dynamic Roman period. Moving into the Early Middle Ages (ca 500–1000 CE), a noticeable increase in the prevalence of R1a is observed, alongside R1b and E1b as the most common haplogroups. While I2a is present in medieval samples, its prevalence is significantly lower than what might be inferred from the contemporary genetic makeup of SEE and is more prominent in the later medieval period. In the early medieval period, when major Slavic migrations occurred, its presence was rather scarce. Also, as mentioned previously, the presence of this haplogroup is identified in samples originating from Copper Age archaeological sites in Croatia, thus challenging the notion that this haplogroup arrived in the region solely through recent migrations during the Middle Ages. This suggests that its existence predates the specified period, although its prevalence according to this initial ancient DNA data may have been considerably lower compared to contemporary frequencies.

On the other hand, R1a does not appear in this sample until Antiquity (only one individual), and its presence became more pronounced in the Middle Ages, giving support to the thesis that this marker can, to a certain extent, be linked to the population movements of Slavs in 6–7th century CE. Data from Olalde and colleagues suggest that individuals harboring the R1a haplogroup were present in SEE before the early medieval Slavic migrations, however, in low occurrence, as its frequency increased from 7% to 20% after the fall of the Roman Empire [19]. 

Similarly, as for R1a, SEE aDNA data confirm the hypothesis that most present-day R1b lineages in Europe derive from a handful of Late Neolithic/Early Bronze Age male founders [45]. This haplogroup was not common in Europe before the Late Neolithic/Bronze Age when its frequency rose, and aDNA R1b individuals are most prevalent in Bronze and Iron Age Croatia while maintaining their high presence during Antiquity and the Early Middle Ages. In the Olalde et al. supplementary data, a significant percentage of 16% was present during Roman and medieval times, which is higher than observed for this haplogroup today (around 10%). The decreased occurrence of this haplogroup nowadays could suggest a significant input of Slavic and other populations in the dynamic Middle Ages period that led to a decline in R1b and an increase in the R1a lineage in SEE [19].

Regarding the E1b haplogroup, our published and unpublished dataset of ancient DNA Croatian samples shows that this haplogroup was present in SEE already in the Neolithic period, although in low frequency. Its presence increased in Antiquity, as well as in the Early Middle Ages. Similar to the R1b clade, its current prevalence of around 10% in the modern Y-chromosome pool in SEE can be explained by the influx of populations from Central Europe that led to its general decline in the region.

Our ancient DNA evidence for Neolithic markers’ presence in SEE points to the same interesting pattern. Namely, the prevalence of the G2a lineage in modern SEE is considerably lower than its frequency and distribution among more ancient populations from this area—it was highly present in SEE and other European regions during the Neolithic, Bronze Age, and Iron Age [11,13,19,57]. This concurs with our unpublished SEE data, where its presence is expectedly significant during the Neolithic, with a decreasing trend in Antiquity and the Early Middle Ages. In their latest study from 2022, Lazaridis and colleagues assert that the medieval Slavic migrations significantly impacted the region, leading to a notable decline in Anatolian Neolithic ancestry within Southeastern Europe [14].

## 4. Future Developments in Y-Chromosome Mapping of Southeastern Europe

In the past decade, advancements in the study of ancient DNA extracted from skeletal remains have provided updated insights into the demographic history of SEE, which has raised new questions. We are currently reexamining previously proposed migration routes and time frames of human settlement in this region. Because modern Croatian genetic data stem from a sample tailored for population genetics research, while ancient DNA analyses rely on samples from archaeological rather than population genetic studies, it is imperative to broaden the sample size for population analysis of ancient DNA from the designated area to its maximum extent. To answer the above-elaborated question, deeper ancient DNA analysis of Copper Age and Iron Age sites, including a wider temporal context (e.g., Late Middle Ages), and more extensive population-scale ancient DNA studies, would be needed, as well as their interpretation in an interdisciplinary population genetic framework.

## Data Availability

This study did not generate or analyze any new data. Therefore, data sharing is not applicable to this article.
